# The Development and Validation of the Doctoral Student Identity Scale

**DOI:** 10.3389/fpsyg.2021.688948

**Published:** 2021-12-07

**Authors:** Jia-lu Zhao, Fu Chen, Xiao-ming Jia

**Affiliations:** ^1^Center for Counseling and Psychological Development Guidance Center, Tsinghua University, Beijing, China; ^2^School of Humanities and Social Sciences, Beijing Institute of Technology, Beijing, China; ^3^Faculty of Education, University of Macau, Macao, Macao SAR, China

**Keywords:** socialization theory, doctoral student identity, scale, doctoral student, exploratory factor analysis (EFA), confirmatory factor analysis (CFA)

## Abstract

**Objective:** Based on how the identity of doctoral students is recognized and understood in the context of Chinese culture, we developed a doctoral identity scale using both qualitative and quantitative analyses.

**Methods:** The initial project of the Scale was formed through qualitative analyses and expert consultation. Nine hundred and ninety-one doctoral students were officially tested, and 982 valid questionnaires were obtained. They were randomly divided into two parts, and 491 of which were assessed for item Response Theory (IRT) and exploratory factor analysis (EFA) and 491 of which were assessed for confirmatory factor analysis (CFA). The Subjective Well-Being Scale (SWB), the Rosenberg Self-Esteem Scale (RSE), and the Psychological Sense of School Membership Scale (PSSM) were used to test its the criterion-related validity. One hundred and forty-one students were selected for retesting after 8 weeks.

**Results:** The doctoral student identity questionnaire consisted of two factors identity exploration and identity commitment, explaining 57% of the total variance. The results of CFA showed that the two-factor model fitted the data well. The two dimensions of the Doctoral Student Identity Scale were significantly and positively correlated with the two dimensions of the SWB scale (0.32–0.66), the latent factor of the RSE scale (0.42–0.55), and the latent factor of the PSSM scale (0.52–0.62). Composite reliability values for exploration and commitment were 0.79 and 0.83 respectively, and the values of McDonald’s omega for exploration and commitment were 0.81 and 0.85 respectively. The test-retest reliability of the total questionnaire was 0.842.

**Conclusion:** The Doctoral Student Identity Scale was developed with good reliability and validity, and can be used as a reliable tool for measuring the doctoral student identity. In addition, the questionnaire will provide corresponding ideas and methods for studying the identity issues of specific groups.

## Introduction

Doctoral student identity development is a crucial dimension of the doctoral student experience (Leshem, 2020). Doctoral students possessing a good sense of identity is an important indicator of the development level of higher education and the academic support of doctoral students receive, and it is helpful as a measure of their career development prospects and for tracking of their ability changes (Green, 2005). Doctoral student identity will increase the enthusiasm of the individual for work and study, which is beneficial for the cultivation of their ability. For example, Chen and Aryee (2007) found that the sense of identity can positively predict the individual’s innovative behavior, and the innovative ability is an important indicator to measure the quality of doctoral education. Many scholars have shown that Doctoral student identity development could be a critical factor in the students’ route in doing a doctorate (Jazvac-Martek, 2009; Baker and Pifer, 2011). Yonghong et al. (2019), who come from China has also proved that the sense of identity of doctoral students can positively predict their innovative ability. Another study found that the life structure, responsibilities, and success expectations of doctoral students are significantly different from those of undergraduate or master’s degree students (Nature, 2018). The doctoral student identity is an important factor influencing doctoral students’ mental health, academic persistence, professional recognition, and academic investment (Kanfer and Ackerman, 1989; Austin, 2002; Foot et al., 2014; Zhao and Jia, 2020). At present, the expansion of enrollment of doctoral education in China continues to expand. According to statistics from the Ministry of Education in 2020, 105,200 students were expected to be enrolled in doctoral programs and 424,200 doctoral students will be enrolled in 2019. In 2020, the enrollment scale was expected to exceed 110,000 (Chinese Education Department, 2020). China is second only to the United States in the expansion of doctoral training. Therefore, it is a very important research content to explore the doctoral student identity.

However, the academic circles have different understandings about the concept of doctoral student identity. Existing research shows that the identity of doctoral students is mainly related to their roles. The socialization theory of doctoral students suggests that the doctoral student identity is one dimension of the socialization of doctoral students. Forming this identity involves a process of role acquisition. Doctoral students develop from having ideal expectations of professional roles at the beginning to becoming members of this professional field; that is, they develop from laymen to insiders (Weidman et al., 2001). This process has gone through the process of identity transformation from “senior student” to “quasi-researcher” to “junior researcher.” Other scholars believe that doctoral students have undergone many status changes during the learning process, first becoming a doctoral student, then a doctoral candidate, then a young scholar, and finally a teacher (Austin, 2002). The research of Kovalcikiene and Buksnyte-Marmiene (2015) shows that the doctoral student identity includes the role of researcher, teacher, and practitioner. More scholars believe that doctoral student identity is mainly the identity of researchers’ role that the development of professional scholarship is an indispensable task for doctoral students (Austin and McDaniels, 2006; Leshem, 2020). According to Austin and McDaniels (2006), developing an identity as a professional scholar is an essential task for a doctoral student. In these studies, the doctoral student identity refers to the roles and identities played by doctoral students, rather than an independent identity.

However, in the Chinese cultural tradition, identity has two meanings. One refers to the identity and appellation of an individual’s position and status in society, and the other refers to the identity of being similar to or different from others (Jiang et al., 2012). For doctoral students, it is an important concept of identity in itself. It is not only concerned about who they will become in the future, but who they already they are (Zhao and Jia, 2020). In the eyes of many Chinese, “becoming a Ph.D. student you are already a Ph.D.” It has nothing to do with the major, and it has nothing to do with the process of Ph.D. The doctoral student itself becomes a “label,” a unique identity, not an identification of the various roles of doctoral students. The reasons mainly include three aspects. One is that being doctoral students means higher social status. Chinese culture emphasizes the value of fame (Yang, 2006). From the perspective of social stratification in ancient China, there are mainly four levels: SHI (scholars), agriculture, industry, and business. “SHI” refers to scholars who gained fame through imperial examinations. The imperial examination system was a system of selecting officials through examinations in ancient China. In the traditional culture of Confucianism, “books have their own golden house,” A poem in the Northern Song Dynasty in China reads “Burnished inferior, only reading high.” This means that all professions are inferior, and only reading to be an official is the right way. These concepts dominate generation after generation. The second is that doctoral students have a respectable social status. The enlightenment bibliography for children in the Ming Dynasty in China, “Zeng Guang Xian Wen,” stated that “the treasure of Senai is the treasure of the country, and Confucianism is the treasure of Xi Shangzhen.” This means that a person who reads is a treasure in the country, and a person who understands etiquette and justice is a pillar of the country. There is also an old saying that “no one knows about 10 years of cold windows, and you become famous in one fell swoop.” In the inheritance of these cultures, scholars as a social identity are respected and admired by the world, which provides an important foundation for understanding the identity of doctoral students in the context of Chinese culture. As the highest level of academic education, doctoral students are even more respected in today’s society. The development of economy and society and the demand for talents make doctorate students a rare resource, and their important role in the acquisition of personal social status and social mobility has become increasingly prominent. The third is that to be doctoral students can bring glory to the family. The Chinese proverb says “ever the dog swaggers when its master wins favor.” This means that when a man gets to the top, all his relations get there with him. In traditional Chinese society, the individual, the family, and the village are one, and the glory of the individual is also the glory of the collective. This is the traditional Chinese concept of “glorious lintel” (King and Myers, 1977). When many people become doctoral students, they are already glorious ancestors and lintels. It can be seen that in the context of Chinese culture, doctoral students themselves have their own identity characteristics, which symbolize and represent a certain social status, and are a special identity that should be redefined and measured.

Redefining the doctoral student identity should return to the earliest understanding of identity. Erikson emphasized that identity is integrated belongingness at the physical and psychological levels (Erikson, 1959). Marcia believed that individuals should participate in an exploration process when identifying meaningful identity commitments. Therefore, exploration and commitment are the core components of identity development. In Marcia’s (1966) conceptual system, the “exploration” refers to people considering different choices, striving to find suitable goals, values and beliefs so as they make meaningful choices; the so-called “commitment” refers to the individual’s make personal investment and self-sacrifice for specific goals, values, and beliefs. In the field of sociology, identity is viewed as a person’s perception of which groups he or she belongs to Deaux (1993). Through the qualitative research of this article, doctoral student identity is defined that the confirmation of the identity of “doctoral students” and the perception of the group to which they belong, as well as the emotional and value meaning of this identity to themselves.

For the measurement of doctoral student’s identity, Kovalcikiene and Buksnyte-Marmiene (2015) jointly compiled a Doctoral Student Identity Scale. The scale covers three scales, namely the researcher’s professional role identity scale, the teacher’s professional role identity scale, and the practitioner’s professional role identity scale. However, this is inconsistent with the understand of the identity of doctoral students in this study, and needs to be revised. In the past, measurement tools based on specific identities mainly focused on ethnic identities. Phinney and Ong (2007) has compiled a Multi group Ethnic Identity Measure (MEIM), which measures the three dimensions of identification: exploration, commitment, and affirmation, and finally formed a revised version of the MEIM with six items. Umaña-Taylor and Fine (2004) compiled the Ethnic Identity Scale (EIS) for 17 items on the basis of MEIM-R. In 2015, it simplified the scale to nine questions (Derlan et al., 2015). Parham and Helms (1981) compiled the Racial Identity Attitudes Scale-Black (RIAS-B), after which Cross and Vandiver (2001) compiled the Cross Racial Identity Scale (CRIS). These measurement tools are closer to the understanding of identity in this study, and can provide support and reference for the compilation of the doctoral identity scale.

In summary, although there is a consensus on the important role of the doctoral student identity, the current research does not consider doctoral students as possessing a special identity. In the context of Chinese culture, the concept of doctoral student identity is different from previous studies, and a new measurement tool is needed to understand the status and characteristics of the development of Chinese doctoral student identity. This article takes full-time academic doctoral students as the research object, explores the structural dimensions of the doctoral student identity, and creates a tool to measure this identity through a scale with a stable structure and good reliability and validity.

### Development and Refinement of the Doctoral Student Identity Scale

#### Item Source: Qualitative Research on the Doctoral Student Identity

##### Aim

Through the qualitative research of interview to understand the understanding of Chinese doctoral student identity of doctoral students and the doctoral student identity how to be developed and obtained.

##### Participants

A combination of purpose sampling and convenience sampling was used to obtain nine doctoral students who passed their Ph.D. thesis defense as interview subjects. Age range is 24–37 years. The basic situation of the sample is shown in Table 1. Before the formal interview, he introduced the research purpose, procedures, confidentiality and other issues, and signed the informed consent.

**TABLE 1:** Basic situation of the sample.

**Item**	**Classification**	**Frequency**	**Item**	**Classification**	**Frequency**
Gender	Male	4	Training method	Unified enrollment	7
	Female	5		Commission orientation	2
Grade^*^	3	2	Marital status	Unmarried	5
	4	5		Unmarried	3
	≥5	2		Married with children	1
Major	Science and engineering	3	Place of residence	Big city	2
	Social science	4		Small and medium cities	1
	Economics and management	1		Town	3
	Art	1		Countryside	3

Grade*: the school system of doctoral students in different universities is different, generally 3–5 years, but can also be extended for 1–2 years.

##### Instrument

The semi-structured interview investigated three main topics:

1. How do you view the identity of a doctor?
2. How do you see yourself as a Ph.D. student?
  (1) When did you start and what did you do that would make you really feel like a Ph.D. student? (Behavior)
  (2) What does it mean to you to become a Ph.D.? (Cognition)
  (3) Do you like to be called a doctor or a doctoral student? What impact will it have on your life? (Emotion)
3. How do you think others perceive you as a Ph.D. student, and how do you feel or think about it?

##### Procedures and Data Analysis

One-to-one interviews were conducted with nine doctoral students, and the interviews were recorded. This research uses categorization analysis to analyze data. First, the analyst verbatim transcribes the audio recordings obtained from the interview. Transcription is an important part of controlling the quality of qualitative research (Steinke, 2004). When transcribing, the analyst strives to be authentic, completely and accurately recording the words, pauses and tone of the interviewee. After the transcription is completed, a transcribed text with a total number of 103,000 Chinese characters is obtained. Then, referring to the suggestions of Braun and Clarke (2006, 2012), the analyst uses the following steps to analyze the data: (1) Read the full text verbatim. While reading, the analyst records some initial thoughts about coding. (2) Create initial codes for the text, and get a total of 2,367 codes. (3) Further summarize the initial codes, discover potential logical relationships, and obtain a total of seven secondary codes. (4) The analyst discussed the secondary codes with another author of this research JXM, a psychologist, and referred to the existing research theories, and finally determined the core codes. After many discussions, the two authors of this article have reached a high degree of agreement on the division of topics. (5) Send the theme obtained in this research and the definition and description of the theme to people who are not in this group to be auditors to avoid major deviations in the entire research.

##### Results

This research has a new definition of the doctoral student identity. Doctoral student identity refers to the confirmation of the identity of the “doctoral student” and the perception of the group to which the doctoral student belongs, as well as the emotional and value meaning of this identity to oneself. Under this definition, a total of seven secondary codes have been obtained through categorization analysis: behavior exploration, value exploration, emotional exploration, meaning, belonging, certainty, and mission. In the determination of core categories, on the one hand, referring to existing research, Phinney and Ong (2007) ’s multi-ethnic identity measurement divides identity into three dimensions: exploration, commitment, and affirmation. On the one hand, there are full discussion within the research team, behavior exploration, value exploration, and emotional exploration can be integrated into identity exploration; meaning, belonging, certainty, and mission can be integrated into identity commitments, and finally two core codes are obtained. These also are two dimensions of the scale. Doctoral student identity exploration refers to the efforts of doctoral students to find suitable goals, values, etc. in the process of obtaining identity, and invest in behavior in this process, experience emotions and make meaningful choices, and include behavior exploration, value exploration, and emotional exploration. Doctoral student identity commitment refers to the stable inner tendency of doctoral students to have a positive attitude and affirmation toward their own group/identity and include meaning, belonging, certainty, and mission. The specific content is shown in Table 2.

**TABLE 2:** Qualitative research results and project examples of doctoral students’ identity.

**Initial codes**	**Secondary codes**	**Core codes**
I have spent time trying to find out more information about the life of a Ph.D. student, such as the benefits of being a Ph.D. student, the procedures in the laboratory, and the way to publish papers.	Behavior exploration	Identity exploration
I think about how people around me view my Ph.D. student.	Value exploration	
During my Ph.D. study, I will experience anxiety, loneliness, and confusion.	Emotional exploration	
The process of studying a Ph.D. has broadened my horizons and greatly changed my outlook on the world and life.	Meaning	Identity commitment
I have a strong sense of belonging to the doctoral student group.	Belonging	
I understand pretty well what being a doctoral student means to me.	Certainty	
I have a sense of mission in the professional field and in my academic development.	Mission	

### Establish a Preliminary Scale

According to the qualitative research results, the two core codes of identity exploration and identity commitment are used as the two dimensions of the Doctoral Student Identity Scale, and the project pool is developed on seven secondary codes. The first step is to select the initial coded text corresponding to the seven sub-dimensions to compile the preliminary test scale items, a total of 70 items; the second step, refer to the items of the multi-group ethnic identity test (MEIM-R) (Phinney and Ong, 2007), the research team analyzed the content of the scale items and eliminated vague statements, and then classified the items measuring similar structures into the same group. In the same group of projects, the projects with the same meaning were merged into one project. After preliminary evaluation, a project library of 35 projects was formed. In the third step, using the Delphi method, a panel of 11 domain experts was invited to further evaluate the quality of each item. Considering the representativeness, authoritativeness, and expertise of the experts, we invited a panel of experts with the following qualifications. First, the experts had worked as a researcher or practitioner in education or psychology for more than 20 years; they had obtained a senior professional title or equivalent (e.g., full professor); and they were available to participate throughout the study. After it was evaluated by the expert panel, the item pool was refined to 12 candidate items.

## Method

### Participants and Procedure

Data were collected from a sample of 991 doctoral students using both convenience and snowball sampling. Specifically, we distributed an online survey to doctoral students from different colleges in China through social networks and websites specific to doctoral students. In addition, we asked participating students and their coworkers to identify other potential participants from their acquaintances. To improve the data quality, we conducted preliminary data screening to remove questionable responses. Specifically, we used a validity check item to identify and remove participants who did not select a specific response. In addition, we removed participants with the behavior of straight lining, namely providing the same response for all items. To detect straight lining, we examined each participant’s responses for a lack of response variability indicated by that all items contained the same response. In other words, a participant with all items associated with the same response would be removed for data analysis. The preliminary data screening resulted in a final sample of 982 doctoral students, which was split into two subsamples (A and B). Subsample A consisted of 491 students (228 female) with a mean age of 29.67 years (SD = 5.64) and was used as the exploratory sample. Subsample B consisted of 491 students (221 female) with a mean age of 29.67 years (SD = 5.48) and was used as the confirmatory sample.

Eight weeks after the first survey, we randomly collected data to examine test-retest reliability through a follow-up online survey distributed to 141 participants who completed the first survey. The data screening for the follow-up survey eliminated responses from six participants, resulting in a final test-retest sample of 135 students. Moreover, we used cluster sampling to collect data from 277 doctoral students from a college in Beijing, China, for a validation sample. In addition to completing the Doctoral Student Identity Scale, participants in the validation sample completed surveys of measures for evaluating concurrent validity (see below). After data screening, the final validation sample consisted of 265 participants (107 female) with a mean age of 27.81 years (SD = 0.64).

### Measures

Multiple studies have also shown that identity has been correlated with numerous psychological adjustment and well-being measures, including self-esteem, subjective well-being (SWB), sense of belonging (Yip and Fuligni, 2002; Lee, 2003; Lee and Yoo, 2004; Phinney and Ong, 2007). In addition, in the field of sociology, identity is viewed as a person’s perception of sense of belonging in a group (Deaux, 1993). We, therefore, examined the relationship between doctoral student identity and three relevant measures for criterion related validity: SWB, self-esteem and sense of school membership.

#### Doctoral Student Identity

The doctoral student identity consisted of eight items, respectively, rated on a five-point Likert scale (1 = strongly disagree, 2 = disagree, 3 = neither disagree nor agree, 4 = agree, and 5 = strongly agree). Cronbach’s alpha for this scale is presented in the results.

#### Subjective Well-Being

The SWB scale, developed by Diener and Emmons (1984) and adapted by Xu and Qiu (2005), consists of 19 items measuring two components of well-being: affective and cognitive dimensions. The scale is rated on a seven-point Likert scale (1 = strongly disagree and 7 = strongly agree). Cronbach’s alpha was 0.85 for this study. A higher value on each subscale indicates a stronger level of each well-being component.

#### Rosenberg Self-Esteem Scale

The Rosenberg self-esteem (RSE) scale is a unidimensional self-esteem measure consisting of 10 items. The scale is rated on a five-point Likert scale (1 = strongly disagree and 5 = strongly agree). Cronbach’s alpha was 0.74 for this study. A higher value on the scale indicates higher self-esteem.

#### Psychological Sense of School Membership Scale

The Chinese version of the Psychological Sense of School Membership (PSSM) scale, revised by Pan et al. (2011), consists of 18 items measuring students’ sense of school membership. The scale is rated on a five-point Likert scale (1 = never and 5 = always). Cronbach’s alpha was 0.74 for this study. A higher value on the scale indicates a stronger level of sense of school membership.

### Statistical Analysis

In addition to conventional descriptive and reliability statistics, we used item discrimination and item information provided by Item Response Theory (IRT) analysis for item selection and item deletion using the data of subsample A. After selecting items by descriptive and IRT analyses, we conducted exploratory factor analysis (EFA) to examine the underlying factor structure of the scale. Using subsample B, we conducted confirmatory factor analysis (CFA) to validate the potential factor structure identified by EFA. Moreover, we evaluated the psychometric properties and validity of the scale using both subsample B and the validation sample. No extreme outliers were detected for both subsamples based on the Mahalanobis distance.

The following model-data fit evaluation indices were used for CFA: the comparative fit index (CFI), the Tucker Lewis index (TLI), the root mean square error of approximation (RMSEA), and the standardized root mean square residual (SRMR). CFI and TLI values range from 0 to 1, and a value close to 1 indicates good fit (Wang and Wang, 2012). Earlier convention was to use 0.90 as a cutoff value for an acceptable fit (Bentler and Bonett, 1980). More recently, however, Hu and Bentler (1998, 1999) suggested a cutoff value of 0.95. For RMSEA, Browne and Cudeck (1992) suggested that values ranging from 0.05 to 0.08 indicate a fair fit, while it was suggested that a cutoff value of 0.08 should be used to signify a good fit more recently (Hu and Bentler, 1999; Marsh et al., 2004). However, it should be noted that these criteria cannot be used as “golden rules” as they were validated with limitations (Marsh et al., 2004). Finally, it should be noted that Chi-square statistics are not often used to evaluate model-data fit given that they are very sensitive to large samples (Wang and Wang, 2012). There were no missing data in this study. Data analyses were conducted using R (R Core Team, 2018), and factor analysis was implemented using the lavaan package (Rosseel, 2012) with maximum likelihood estimation with robust standard errors as the estimator.

## Results

### Scale Construction

#### Descriptive Item Analysis for Item Deletion

Table 3 presents the descriptive summary of each item in the scale. The normality analysis showed that item 12 had a large kurtosis value, indicating that the majority of respondents selected the same response for item 12; therefore, normality was not met for item 12. In terms of item-total correlation, according to the convention guidelines in the literature (e.g., Boateng et al., 2018), no items were considered problematic given that all items showed an item-total correlation above 0.3 (ranging from 0.39 to 0.71). Regarding interitem correlation (see Table 4), for items measuring factor 1 (items 1–6), the average interitem correlation was 0.44, and items 5 and 6 showed relatively lower intercorrelations with most of the other items. In addition, dropping item 5 or item 6 would increase the average interitem correlation but would not largely decrease Cronbach’s alpha. For items measuring factor 2 (items 7–12), the average interitem correlation was 0.51, and all items showed a desirable intercorrelation above 0.3 (Boateng et al., 2018). However, dropping item 12 would largely increase the average interitem correlation but would not substantially change Cronbach’s alpha. These results suggested that items 5, 6, and 12 were potentially problematic and should be deleted from the preliminary scale.

**TABLE 3:** Descriptive summary of the Doctoral Student Identity Scale (*n* = 491).

**Item ID**	**M**	**SD**	**Skew**	**Kurtosis**	**SE**
Item 1	3.72	0.95	−0.68	–0.09	0.04
Item 2	3.75	0.91	−0.86	0.36	0.04
Item 3	3.92	0.83	−1.02	1.34	0.04
Item 4	3.91	0.83	−0.96	1.29	0.04
Item 5	3.21	1.10	−0.40	–0.63	0.05
Item 6	3.84	0.87	−0.99	1.13	0.04
Item 7	3.57	0.95	−0.50	–0.05	0.04
Item 8	3.80	0.87	−0.76	0.80	0.04
Item 9	3.54	0.95	−0.60	–0.07	0.04
Item 10	3.58	0.96	−0.47	–0.10	0.04
Item 11	3.79	0.88	−0.92	0.98	0.04
Item 12	4.12	0.77	−1.08	2.21	0.03
					

**TABLE 4:** Polychoric correlation matrix for the Doctoral Student Identity Scale (*n* = 491).

**Items**	**1**	**2**	**3**	**4**	**5**	**6**	**7**	**8**	**9**	**10**	**11**
2	0.69										
3	0.59	0.59									
4	0.42	0.47	0.55								
5	0.26	0.32	0.24	0.36							
6	0.28	0.32	0.33	0.64	0.51						
7	0.37	0.31	0.39	0.42	0.58	0.54					
8	0.34	0.31	0.39	0.53	0.39	0.52	0.67				
9	0.36	0.34	0.38	0.41	0.52	0.43	0.75	0.66			
10	0.29	0.21	0.28	0.31	0.27	0.30	0.55	0.49	0.52		
11	0.25	0.30	0.37	0.36	0.34	0.40	0.52	0.48	0.51	0.63	
12	0.31	0.35	0.39	0.45	0.35	0.45	0.44	0.44	0.45	0.34	0.55

*All correlation coefficients are significant at the 0.001 level.*

#### Item Response Theory Analysis for Item Deletion

Prior to the IRT analysis, we evaluated the unidimensionality of each subscale. For factors 1 and 2, the eigenvalue of the first factor was much larger than the eigenvalue of the second factor (five times larger, 2.63 vs. 0.48, and 12 times larger, 3.19 vs. 0.27, respectively). This indicated that the variances of the item scores for both factors could be accounted for by a major dimension, and therefore, the unidimensionality for both factors was met. Regarding the local independence assumption, basic local item independency can be revealed by that items measuring the same factor were significantly correlated with other, but the majority of correlation coefficients were below 0.50 (see Table 2). In addition, factor analysis for each dimension indicated that item residuals were not or weakly correlated with each other with a correlation coefficient below 0.20 except a correlation coefficient of 0.23 between items 5 and 6. In general, the local item independence assumption was met for the IRT analysis. In addition, we also examined the monotonicity assumption using the Mokken scale analysis to produce the item scalability coefficient for each item. The coefficients range from 0.34 to 0.49, indicating a weak to moderate monotonicity for each item (Sijtsma and Molenaar, 2002).

**FIGURE 1:**
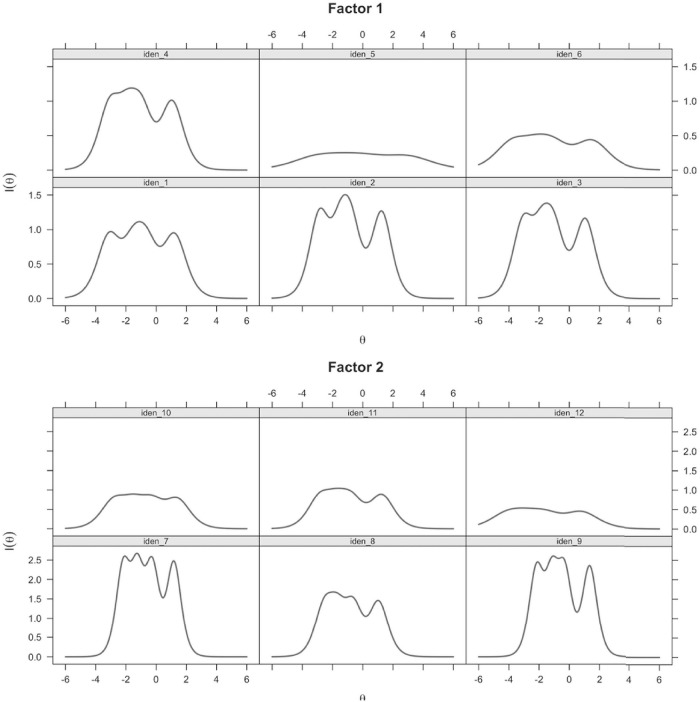
Item information curves for the Doctoral Student Identity Scale.

The IRT analysis was conducted with the graded response model for both factors. The results for factor 1 showed that all items except items 5 and 6 were characterized by high item discrimination (see Table 5), indicating that they were good indicators of the latent construct of factor 1. According to the item information curves (see Figure 1), items 5 and 6 showed low information for any range of the latent construct level, indicating that these two items could not be used to accurately measure respondents’ latent traits on factor 1. For items of factor 2, all items except item 12 showed high item discrimination (see Table 5), indicating that they were good indicators of the latent construct of factor 2. According to the item information curves (see Figure 1), compared with other items, item 12 showed lower information for any range of the latent construct level (an almost flat information curve), indicating that item 12 contributed no information to the measurement of factor 2. In summary, given the IRT analysis results, items 5, 6, and 12 were potentially problematic and were deleted from the preliminary scale.

**TABLE 5:** Item parameters estimated by the graded response model for the Doctoral Student Identity Scale (*n* = 491).

	**a**	**b1**	**b2**	**b3**	**b4**
Item 1	1.93	−3.11	−1.46	−0.71	1.21
Item 2	2.24	−2.85	−1.42	−0.85	1.25
Item 3	2.15	−3.04	−1.79	−1.09	1.05
Item 4	2.00	−3.05	−1.90	−1.03	1.05
Item 5	0.90	−2.99	−1.37	0.12	2.93
Item 6	1.31	−3.72	−2.19	−1.18	1.48
Item 7	3.13	−2.17	−1.25	−0.27	1.17
Item 8	2.39	−2.51	−1.71	−0.65	1.04
Item 9	3.06	−2.16	−1.14	−0.33	1.34
Item 10	1.75	−2.78	−1.53	−0.29	1.39
Item 11	1.86	−2.80	−1.76	−0.81	1.26
Item 12	1.32	−4.03	−2.98	−1.66	0.79

*A refers to the item discrimination parameter, and b1–b4 refer to the location parameter for scores 1–4.*

#### Exploratory Factor Analysis

Exploratory factor analysis was conducted on the data of the items excluding items 5, 6, and 12. Prior to the EFA, we evaluated the suitability of the data for EFA. Specifically, the KMO measure of sampling adequacy was 0.85, above the widely used cutoff value of 0.5, and Bartlett’s test of sphericity was significant, χ^2^(36) = 2136.04, *p* < 0.001, indicating that EFA was suitable for all nine items. Given that the normality for some items was not met, we used principal axis factoring for EFA. We used parallel analysis and the Very Simple Structure (VSS) criteria to determine the number of factors. Although the parallel analysis suggested a four-factor structure, the eigenvalues of the third and fourth factors were 0.14 and 0.08, respectively, which were far below the criterion of 1. Moreover, the VSS analysis showed that VSS complexity 1 achieved a maximum of 0.79 with one factor and VSS complexity 2 achieved a maximum of 0.75 with two factors. The Velicer’s Minimum Average Partial (Velicer, 1976) achieved a minimum of 0.05 with two factors. Therefore, taking into account the results from both analyses, we adopted a two-factor model for EFA. The EFA results showed that the two factors explained 57% of the total variance in the item scores. According to the factor loadings (see Table 6), items 1–4 loaded on factor 1 (named “exploration”), and items 7–11 loaded on factor 2 (named “commitment”). No items demonstrated cross-loadings or factor loadings below 0.4. The two factors were moderately correlated with each other (*r* = 0.54).

**TABLE 6:** Exploratory factor analysis factor loadings for the two-factor model of the Doctoral Student Identity Scale (*n* = 491).

**Items**	**Description**	**Exploration**	**Commitment**
1	I have spent time trying to find out more information about the life of a Ph.D. student, such as the benefits of being a Ph.D. student, the procedures in the laboratory, and the way to publish papers.	0.77	0.00
2	I have read books, magazines, or information related to doctoral studies and scientific research life to help me better understand my doctoral career.	0.85	−0.07
3	I have often talked to other people, such as a doctoral supervisor or a senior apprentice, to learn more about my doctoral career.	0.68	0.11
4	I understand pretty well what my doctoral student identity means to me.	0.45	0.28
7	I have a strong sense of belonging to the doctoral student group.	0.00	0.83
8	I understand pretty well what being a doctoral student means to me.	0.07	0.73
9	I feel a strong attachment to the doctoral student group.	0.02	0.79
10	I feel very strong sense in my heart that I can be called a doctoral student.	−0.08	0.74
11	I have a sense of mission in the professional field and in my academic development.	0.01	0.68

#### Confirmatory Factor Analysis

Confirmatory factor analysis was conducted to validate the two-factor structure of the scale identified by the EFA using subsample B. The model showed a poor fit to the data: χ^2^(26, *N* = 491) = 105.54, *p* < 0.001, RMSEA = 0.078, 90% CI (0.066, 0.091), *p* < 0.001, SRMR = 0.060, CFI = 0.925, and TLI = 0.896. The modification indices suggested that items 10 and 11 should be correlated with each other for an improved fit. Given that item 11 had the lowest factor loading on factor 2 and was a new item written by the authors, we revised the two-factor model by dropping item 11 for the CFA. The revised model showed an acceptable fit to the data: χ^2^(19, *N* = 491) = 60.18, *p* < 0.001, RMSEA = 0.066, 90% CI (0.051, 0.082), *p* < 0.001, SRMR = 0.057, CFI = 0.951, and TLI = 0.928. Moreover, all items showed significant and high standardized factor loadings on both factors (0.66–0.76 for exploration and 0.58–0.83 for commitment, see Table 7). Consequently, the EFA and CFA analyses resulted in a final version of the scale consisting of eight items, with items 1–4 and items 7–10 measuring factors 1 and 2, respectively.

**TABLE 7:** Confirmatory factor analysis factor loadings (standard errors) and unstandardized error variances (standard errors) for the two-factor model of the Doctoral Student Identity Scale (*n* = 491).

**Item**	**Unstandardized**		**Standardized**		**Error variance**
	**Exploration**	**Commitment**	**Exploration**	**Commitment**	
1	1.00		0.71		0.40 (0.05)
2	0.98 (0.09)		0.66		0.48 (0.07)
3	1.03 (0.09)		0.76		0.30 (0.04)
4	0.88 (0.11)		0.66		0.40 (0.05)
7		1.03 (0.05)		0.83	0.31 (0.04)
8		0.78 (0.06)		0.72	0.35 (0.04)
9		1.00		0.83	0.28 (0.04)
10		0.70 (0.08)		0.58	0.60 (0.06)

*Values in parentheses are standard errors.*

### Evidence of Validity for the Doctoral Student Identity Scale

In this study, we adopted the contemporary view of validity (Zumbo and Chan, 2014) to collect evidence with statistical techniques to validate the Doctoral Student Identity Scale. Specifically, according to Zumbo (2005), structural equation modeling (SEM) was used to evaluate the score structure (i.e., CFA), the criterion-related validity (using SEM to examine the relationships between the two factors of the scale and three external measures of relevant constructs), and the reliability (internal consistency indicated by the composite reliability) of the scale. Moreover, the test-retest reliability of the scale was also examined in our study.

#### Internal Consistency

Composite reliability and McDonald’s omega were used to measure the internal consistency in the items of the Doctoral Student Identity Scale. Composite reliability indicates “the shared variance among the observed variables used as an indicator of a latent construct” (Fornell and Larcker, 1981). According to Netemeyer et al. (2003), a construct measuring by five to eight items should have a minimum composite reliability of 0.80. In our study, composite reliability values for exploration and commitment were 0.79 and 0.83, respectively. Although the composite reliability for exploration is slightly below 0.80, it should be noted that both exploration and commitment were measured by four items and the magnitude of composite reliability heavily relies on the number of items in a scale. Moreover, the values of McDonald’s omega for exploration and commitment were 0.81 and 0.85, respectively, indicating that the two factors have satisfactory internal consistency.

#### Test-Retest Reliability

Using the follow-up sample, we examined the test-retest reliability of the scale. The Pearson correlation coefficient for the total scores between the two surveys was 0.84, and the correlation coefficients between the two surveys for exploration and commitment were 0.71 and 0.76, respectively, indicating high test-retest reliability of the scale.

#### Score Structure Validity

We used the CFA results to examine the score structure validity of the scale. According to the CFA results, the eight items loaded well on two separate factors without cross-factor loadings, indicating an explicit two-factor model. Moreover, the score structure validity could be further justified by the following correlations: the two factors were strongly correlated with the total scores, with a correlation coefficient of 0.91. All items were moderately to highly correlated, as their corresponding factors with correlation coefficients ranged from 0.63 to 0.89. All items were moderately to highly correlated with the total scores, with correlation coefficients ranging from 0.63 to 0.75. The two factors were moderately correlated with each other, with a correlation coefficient of 0.68. Items measuring a factor were weakly to moderately correlated with the other factor, with correlation coefficients ranging from 0.37 to 0.57. Finally, items measuring different factors were weakly to moderately correlated with each other, with correlation coefficients ranging from 0.22 to 0.50.

#### Criterion-Related Validity

The criterion-related validity of the Doctoral Student Identity Scale was evaluated by a SEM examining the associations of its two latent factors with factors of three external measures related to the doctoral student identity. The results (see Table 8) showed that the two dimensions of the SWB scale were significantly and positively correlated with two dimensions of the Doctoral Student Identity Scale (*r*s = 0.32, 0.52, 0.49, and 0.66, *p*s < 0.001). The latent factor of the RSE scale was significantly and positively correlated with the two dimensions of the Doctoral Student Identity Scale (*r*s = 0.42 and 0.55 for exploration and commitment, respectively, *p*s < 0.001). The latent factor of the PSSM scale was significantly and positively correlated with the two dimensions of the Doctoral Student Identity Scale (*r*s = 0.52 and 0.62 for exploration and commitment, respectively, *p*s < 0.001). These results indicate that the newly developed Doctoral Student Identity Scale correlated well with established and validated measures evaluating similar constructs.

**TABLE 8:** Correlations and regression coefficients between the Doctoral Student Identity Scale factors and the factors of SWB, RSE, and PSSM (*n* = 265).

	**Exploration**	**Commitment**
SWB: affective	0.32	0.52
SWB: cognitive	0.49	0.66
RSE	0.42	0.55
PSSM	0.52	0.62

*SWB, subjective well-being; RSE, Rosenberg self-esteem; PSSM, Psychological Sense of School Membership. All correlation coefficients are significant at the 0.001 level.*

## Discussion

The study introduced the development and verification process of the Doctoral Student Identity Scale. The characteristics of the Doctoral Student Identity Scale are mainly reflected in the following three aspects.

First of all, starting from the concept of doctoral student identity, through interviews and qualitative research analysis, it is found that the existing literature’s understanding of doctoral students’ identity is different from the concept of doctoral students’ identity under the Chinese cultural background. In the existing literature, the identity of doctoral students is based on the understanding of role recognition, and it is believed that the identity of doctoral students is a transitional identity for doctoral students to develop professional identity (Cast, 2003; Leshem, 2017), and the final doctoral student identity is identified different roles, such as the academics, the professional independent scholar and the researcher (McAlpine and Amundsen, 2009; Trafford and Leshem, 2009; Leshem, 2020). These studies believe that doctoral student identity is the recognition of different professional identities, and it can also be a transition from one professional role to another (Hall and Burns, 2009). This research defines the identity of doctoral students as the confirmation of the identity of “doctoral students” and the perception of the group to which they belong, as well as the emotional and value meaning of this identity to themselves. This definition is understood by taking the doctoral student as an independent identity and identification. This is defined based on the understanding of the doctoral student in Chinese culture, and it is a new understanding of the identity of doctoral students.

Secondly, from the point of view of the item sources of the doctoral student identity scale, the scale items mainly come from qualitative research data. The two dimensions of identity exploration and identity commitment obtained from qualitative research results. Phinney’s ethnic identity development theory is based on the three dimensions of exploration, commitment, and recognition (Phinney and Ong, 2007). However, this study found that commitment and recognition are not independent dimensions, and there are many overlapping areas in content. Our research is more consistent with Marcia’s identity state model (Marcia, 1980). However, Marcia’s identity status model is more focused on the behavioral dimension of exploration, but Chinese doctoral students devote much attention to the exploration of values and meanings. With regard to identity commitment, this study not only considers doctoral students’ sense of belonging to their current doctoral student identity but also considers their sense of mission for their future doctoral status. Confucianism advocates social and group concepts such as cultivation, harmony, governance, and world peace. In such a culture, individuals emphasize the “social self,” the “collective self,” and the “public me” (Sun and Ping, 2015). Thus, the identity of Chinese doctoral students is not only a personal identity but also a cultural identity.

Thirdly, in the verification of specific questionnaire items, IRT was used to reflect the relationship between the research subjects and the questions. After the analysis of the IRT model, items 5, 6, and 12 were deleted. According to the EFA and CFA, the loading of item 11 on factor 2 was small. After item 11 was deleted, both factor 1 and factor 2 had a significant and higher corresponding factor loading (factor 1: 0.66–0.76; factor 2: 0.58–0.83). Two factors explained 57% of the total variance of all items, and the correlation coefficient between the two was 0.54. In examining the validity of the standard association, the SWB scale, the RSE scale and the PSSM scale were used to assess the standard variables. The structure showed that the Doctoral Student Identity scale and its various dimensions were significantly positively correlated with the validity of the calibration, indicating that the scale had good calibration-related validity. Reliability analysis showed that the α coefficients of the two factors were 0.77 (exploration) and 0.82 (commitment), indicating that the internal consistency of the two factors of the scale was highly reliable. The test-retest reliability was 0.842, which met the requirements of psychometrics.

There remain a number of methodological limitations that need to be addressed in future studies. For example, the study collected data from Chinese doctoral students, and whether it is applicable to participants of non-Chinese nationality needs further investigation. In addition, we encourage future studies to evaluate the developed scale using longitudinal data as the scale has only been examined using cross-sectional data in the current study.

## Conclusion

The measurement of the doctoral student identity in previous studies was mainly based on the measurement of the identity of doctoral students in different roles. Based on the Chinese society’s understanding of the identity of doctoral students, this research regards doctoral students as an independent identity, and compiled a doctoral identity scale based on qualitative research data. The scale has good reliability and validity, and can provide a reliable tool for measuring the doctoral student identity. In addition, the questionnaire will provide corresponding ideas and methods for studying the identity issues of specific groups.

## Data Availability Statement

The original contributions presented in the study are included in the article/supplementary material, further inquiries can be directed to the corresponding authors.

## Ethics Statement

This study was carried out in accordance with the recommendations of the Ethics Committee of the School of Humanities and Social Sciences at Beijing Institute of Technology with written informed consent from all subjects. All subjects gave written informed consent in accordance with the Declaration of Helsinki. The protocol was approved by the Ethics Committee of the School of Humanities and Social Sciences at Beijing Institute of Technology. The patients/participants provided their written informed consent to participate in this study.

## Author Contributions

J-LZ: study conception and design, interpretation of the data, drafting of the manuscript, and critical revision. X-MJ: acquisition of the data, interpretation of the data, and drafting of the manuscript. FC: analysis and interpretation of the data, and drafting of the manuscript. All authors read and approved the final manuscript.

## Conflict of Interest

The authors declare that the research was conducted in the absence of any commercial or financial relationships that could be construed as a potential conflict of interest.

## Publisher’s Note

All claims expressed in this article are solely those of the authors and do not necessarily represent those of their affiliated organizations, or those of the publisher, the editors and the reviewers. Any product that may be evaluated in this article, or claim that may be made by its manufacturer, is not guaranteed or endorsed by the publisher.
